# Benchmarking machine learning models in lesion-symptom mapping for predicting language outcomes in stroke survivors

**DOI:** 10.3389/fnimg.2025.1573816

**Published:** 2025-05-30

**Authors:** Deepa Tilwani, Christian O'Reilly, Nicholas Riccardi, Valerie L. Shalin, Dirk-Bart den Ouden, Julius Fridriksson, Svetlana V. Shinkareva, Amit P. Sheth, Rutvik H. Desai

**Affiliations:** ^1^Artificial Intelligence Institute, University of South Carolina, Columbia, SC, United States; ^2^Department of Computer Science and Engineering, University of South Carolina, Columbia, SC, United States; ^3^Carolina Autism and Neurodevelopment Research Center, University of South Carolina, Columbia, SC, United States; ^4^Institute for Mind and Brain, University of South Carolina, Columbia, SC, United States; ^5^Department of Communication Sciences and Disorders, University of South Carolina, Columbia, SC, United States; ^6^Department of Psychology, Wright State University, Dayton, OH, United States; ^7^Department of Psychology, University of South Carolina, Columbia, SC, United States

**Keywords:** aphasia, lesion-symptom mapping, neuroimaging, multivariate analysis, stroke, machine learning

## Abstract

Several decades of research have investigated the neural connections between stroke-induced brain damage and language difficulties. Typically, lesion-symptom mapping (LSM) studies that address this connection have relied on mass univariate statistics, which do not account for multidimensional relationships between variables. Machine learning (ML) techniques, which can capture these intricate connections, offer a promising complement to LSM methods. To test this promise, we benchmarked ML models on structural and functional MRI to predict aphasia severity (*N* = 238) and naming impairment (*N* = 191) for a cohort of chronic-stage stroke survivors. We used nested cross-validation to examine performance along three dimensions: (1) parcellation schemes (JHU, AAL, BRO, and AICHA atlases), (2) neuroimaging modalities (resting-state functional connectivity, structural connectivity, mean diffusivity, fractional anisotropy, and lesion location) and (3) ML methods (Random Forest, Support Vector Regression, Decision Tree, K Nearest Neighbors, and Gradient Boosting). The best results were obtained by combining the JHU atlas, lesion location, and the Random Forest model. This combination yielded moderate to high correlations with the two different behavioral scores. Key regions identified included several perisylvian areas and pathways within the language network. This work complements existing LSM methods with new tools for improving the prediction of language outcomes in stroke survivors.

## 1 Introduction

Lesion-symptom mapping (LSM) plays a major role in studying brain-behavior relationships (Bates et al., 2003; Bendfeldt et al., 2012; Burges, 1998; Forkel and Catani, 2018; Karnath et al., 2019; Moore et al., 2023). Specifically, statistical voxel-based lesion-symptom mapping [VLSM; (Bates et al., 2003)] assesses the relationship between brain lesions and specific behavioral deficits on a voxel-by-voxel basis, allowing the identification of brain regions where damage correlates with behavioral impairment. This represents a significant advance compared to the conventional lesion overlap-subtraction approach (Bates et al., 2003). However, as a univariate method, VLSM is limited for assessing the multivariate lesion-symptom relationship (Walker et al., 2011; Kimberg et al., 2007). Traditional VLSM does not consider correlations among neighboring voxels, although these can enhance detection power (Kimberg et al., 2007; Herbet et al., 2015). VLSM often uses dichotomized lesion data (lesion present or absent), resulting in low variance at each voxel. This can limit its ability to predict continuous dependent variables of clinical significance, such as behavioral impairment (DeMarco and Turkeltaub, 2018).

To overcome these limitations, researchers have explored multivariate lesion-symptom mapping (MLSM) methods (DeMarco and Turkeltaub, 2018), combining all voxels into a single model rather than using separate models for each voxel. One MLSM strategy applies support vector regression (SVR) to multivariate lesion data extracted from predefined ROIs to predict binary behavioral outcomes, such as whether or not a patient exhibits spatial neglect (Smith et al., 2013; Zhao et al., 2018).

However, MLSM also has some drawbacks. These include specific statistical challenges, such as uncertainties in (hyper)parameter selection and how these influence solution regularization and computational cost. These limitations impact result interpretation and limit *post-hoc* computation (Pustina et al., 2017; DeMarco and Turkeltaub, 2018). Recent advances in disconnectome-based mapping now trace how lesions disrupt large-scale white-matter networks. Gleichgerrcht et al. (2017) showed that disconnection patterns predict language deficits beyond focal cortical damage, and Thiebaut de Schotten et al. (2020) introduced population-based disconnectome maps to link white-matter disconnection with behavior. *Yet these approaches still depend on normative tractography templates and rarely incorporate subject-specific reorganization. As a result, their explanatory power for chronic stroke remains only partial*. Machine-learning (ML) models have also become prominent in LSM. Billot et al. (2022b) predicted recovery trajectories, while Talozzi et al. (2023) showed that multimodal features can boost prediction accuracy, and Matsulevits et al. (2024) used interpretable networks to localize language-critical regions. *However, most ML studies employ modest sample sizes, limited cross-validation, and sparse hyper-parameter searches, making it difficult to gauge generalizability and to compare algorithms or imaging modalities on equal footing*.

LSM approaches using ML are a promising complement to traditional VLSM or MLSM methods. ML enables the identification of complex relationships between patterns of brain damage and language deficits that traditional univariate VLSM approaches may not capture. Using SVR-MLSM, a combination of two methods (SVR and MLSM), several studies have obtained improved prediction accuracy for behavioral scores, such as the Comprehensive Aphasia Test [CAT; e.g., *r* = 0.59 in Hope et al. (2013) from lesion volumes and *r* = 0.69 in Yourganov et al. (2016) for the Western Aphasia Battery-Revised (WAB-R) Aphasia Quotient (AQ)]. In a comparative study, Ivanova et al. (2021) suggested that both univariate and multivariate LSM have advantages and recommended that both methods should be used in tandem. Halai et al. (2020) conducted a comprehensive study on how key parameters influence brain-to-behavior prediction models for post-stroke aphasia, focusing on four principal language and cognitive dimensions (phonology, semantics, speech fluency, and executive demand). Using multimodal neuroimaging data (T1 and diffusion-weighted imaging) and advanced ML algorithms, the study demonstrated that models using structural T1 features often matched or outperformed those incorporating diffusion data. Predictive accuracy, assessed via cross-validated metrics, achieved Pearson's correlations ranging from *r* = 0.50 to 0.73. While these findings underscore the potential of ML in aphasia research, measures such as the AQ or Philadelphia Naming Test (PNT) scores have not been extensively evaluated, which remain critical for assessing overall aphasia severity and naming abilities.

Aphasia frequently results from strokes specifically affecting language regions in the left hemisphere (Carey, 2016). Several studies have used MRI techniques to explore the left hemisphere's role in stroke-induced aphasia (Fridriksson et al., 2010; Price, 2000, 2012). Behavioral prediction using various neuroimaging modalities separately, such as annotated lesions, fMRI functional connectivity during resting-state (rsFC), and measures derived from diffusion tensor imaging (DTI), such as structural connectivity (SC) based on fiber tracking, mean diffusivity (MD), and fractional anisotropy (FA), may help further identify patterns and relationships that contribute to predicting aphasia severity, language impairments, and recovery potential. Some results suggest that diffusion-weighted data in lesion-based models do not improve the accuracy of regression models (Hope et al., 2018). Using multimodal data with SVR resulted in correlations ranging from *r* = 0.60 to 0.67 for several different behavioral scores: AQ, fluency, auditory comprehension, naming, speech repetition, and spontaneous speech (Kristinsson et al., 2020). Notably, that study did not include cross-validated feature selection, potentially resulting in data leakage, a tendency for overfitting, and over-optimistic performance assessment (Poldrack et al., 2019).

Here, we systematically benchmark all possible combinations of a factorial design with three methodological factors: brain atlas, neuroimaging modality, and ML algorithm. Specifically, we cross every feasible pairing of commonly used atlases (AAL, AICHA, BRO, JHU), five MRI-derived modalities (lesion, SC, rsFC, FA, MD), and six ML models (Linear Regression, Random Forest, SVR, Decision Tree, K Nearest Neighbors, and Gradient Boosting). Performance is assessed using two behavioral measures that capture complementary aspects of post-stroke language function: the AQ and PNT. Our primary aims are i) to identify which atlas-modality pairings best characterize language-related damage and ii) to determine which ML techniques most accurately predict language scores from neuroimaging data.

## 2 Methods

### 2.1 Data acquisition

#### 2.1.1 Participants

This work leverages data previously collected as part of a multi-site stroke aphasia study, including the Center for the Study of Aphasia Recovery (C-STAR) at the University of South Carolina (USC) and the Medical University of South Carolina (MUSC). Institutional Review Boards at both universities approved the study procedures. All procedures adhere to the Declaration of Helsinki. Informed consent was obtained independently from all participants under the supervision of care partners, considering potential comprehension difficulties for some participants. Travel and lodging expenses were reimbursed for participants living more than 35 miles from the data collection site. Our dataset included individuals who suffered a single stroke to the left hemisphere and who received an MRI scan and a behavioral test (PNT: *N* = 191; AQ: *N* = 238). Participants who suffered lacunar infarcts, bilateral strokes, or damage only involving the brainstem or cerebellum were excluded. We enrolled participants with lesions only in the left hemisphere to account for the fact that language functions are primarily localized to the left hemisphere in most people. The average time elapsed since the occurrence of stroke was 39.4 months, ranging from 5.6 to 237.1 months.

#### 2.1.2 Neuroimaging data

##### 2.1.2.1 Behavioral data acquisition

Neuroimaging data and behavioral scores were collected from 2007 to 2019. PNT (Roach et al., 1996) and WAB-R (Kertesz, 2006) were administered by licensed speech-language pathologists as part of a larger language battery. PNT responses were recorded, transcribed, and scored by trained research assistants. Our outcome measures were AQ for the WAB-R and the total number of correct items for the PNT. AQ and PNT scores were highly correlated (*r* = 0.89) as both measures reflect overall aphasia severity. In the assessment of the WAB-R, a maximal cumulative score of 100 points is allocated, which includes several speech comprehension, speech production, and repetition tasks. For the PNT, line drawings or pictures of objects are presented, and the individual is asked to name each object as accurately and quickly as possible. The primary measure is the proportion of correctly named objects.

##### 2.1.2.2 MRI data acquisition

MRI data were gathered with a Siemens 3T Trio System with a 12-channel head coil and a Siemens 3T Prisma fit scanner with a 20-channel coil. Participants underwent two anatomical MRI sequences: i) T1-weighted imaging sequence with an MP-RAGE (magnetization-prepared rapid-gradient echo) [turbo field echo] sequence with voxel size = 1 mm^3^, FOV (field of view) = 256 × 256 mm, 192 sagittal slices, 9° flip angle, TR (repetition time) = 2,250 ms, TI (inversion time) = 925 ms, TE (echo time) = 4.15 ms, GRAPPA (generalized autocalibrating partial parallel acquisition) = 2, and 80 reference lines; and ii) T2-weighted MRI with a 3D sampling perfection with application-optimized contrasts by using different flip angle evolutions (SPACE) protocol with the following parameters: voxel size = 1 mm^3^, FOV = 256 × 256 mm, 160 sagittal slices, variable flip angle, TR = 3,200 ms, TE = 212 ms, and no slice acceleration. The same slice center and angulation were used as in the T1 sequence. Functional connectivity was measured using resting-state scans. fMRI volumes (196 per participant) were acquired with an echo-planar imaging sequence with FOV = 208 × 208 mm, 64 × 64 matrix size of 3.25 mm isotropic voxels, 75° flip angle, 34 axial slices (3 mm thick with 20% gap yielding 3.6 mm between slice centers), TR = 1,850 ms, TE = 30 ms, GRAPPA = 2, 32 reference lines, and sequential descending acquisition. DTI was captured with a monopolar sequence with 82 isotropic (2.3 mm) volumes ( × 10 B = 0, × 72 B = 1,000), TR = 4,987 ms, TE = 79.2 ms, 90 × 90 matrix, with parallel imaging GRAPPA = 2, and 50 contiguous slices. The sequence was acquired in two series (41 and 43 volumes in each series) with opposite phase encoding, allowing us to correct for spatial distortion using the TOPUP method (Andersson et al., 2003).

#### 2.1.3 Preprocessing

**Figure 1:**
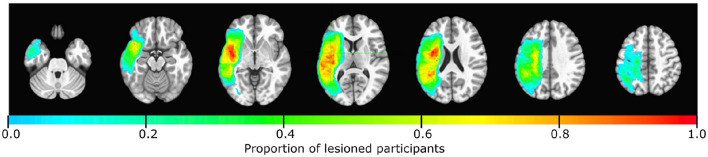
Lesion distribution among the 191 participants is represented using a color-coded scale, where light blue indicates regions with low lesion overlap and red indicates regions with high lesion overlap. The region around the perisylvian fissure showed the highest concentration of overlapping lesions across participants.

Preprocessing is required to normalize spatial scales, correct for motion and noise, and standardize lesion size, among other considerations. Generic preprocessing steps were used for all modalities, followed by additional modality-specific preprocessing steps (see subsections below). Data preprocessing was conducted with Matlab (R2017b, The MathWorks, Inc., Natick, MA) using the nii_preprocess software (https://github.com/neurolabusc/nii_preprocess). This image-processing pipeline was tailored explicitly for clinical stroke populations and is open-source. The nii_preprocess pipeline incorporates scripts to handle diverse MRI data modalities. Its output is transformed into the MNI standard space. The quality of the resulting preprocessed data was validated by visual inspection. In Figure 1, the distribution of lesions among participants is shown.

##### 2.1.3.1 Lesion preprocessing

Lesions were defined by a neurologist (L. Bonilha) using T2-weighted images in MRIcron, a cross-platform viewer for NIfTI images (Rorden et al., 2012). T2-weighted images were co-registered to match the T1-weighted images. Images were then warped to standard space using an enantiomorphic (Nachev et al., 2008) segmentation-normalization (Ashburner and Friston, 2005) custom Matlab script (https://github.com/rordenlab/spmScripts/blob/master/nii_enat_norm.m) to warp images to an age-appropriate template in an SPM-based clinical toolbox (Rorden et al., 2012). Normalization parameters were used to re-slice the lesion into standard space using linear interpolation, with subsequent lesion maps stored at 1 mm isotropic resolution and binarized using a 50% threshold. To avoid the fractional values resulting from interpolation, this step categorizes each voxel as lesioned or not without biasing the overall lesion volume. Normalized images were visually inspected for quality control.

##### 2.1.3.2 fMRI preprocessing

Motion correction for fMRI data was achieved via the SPM12 (Ashburner et al., 2021) *realign and unwarp* default procedure. Slice timing correction was achieved with SPM12. Brain extraction was performed with the default SPM12 *pm_brain_mask function*. The fMRI volume of each subject was aligned to the extracted T2-weighted image to determine the spatial transformation between the fMRI data and the lesion mask. The fMRI data were spatially smoothed with a 6 mm full width at half-maximum Gaussian kernel. Lesion artifacts were eliminated using the process outlined in Yourganov et al. (2017). FSL MELODIC was used to decompose the data into independent components to eliminate potential confounding effects from the lesion on fMRI and calculate the z-scored spatial maps for each component. The maps were then thresholded at *p* < 0.05 and compared with the lesion mask for that patient. If the overlap (measured via the Jaccard index) between the lesion and the thresholded independent component map was more than 50%, the corresponding component was considered to overlap significantly with the lesion. These components were then regressed from the fMRI data using *fsl_refilt* from the FMRIB Software Library (FSL). This ensures that measures derived from the fMRI signals are not unduly influenced by spurious correlations with fMRI signals in lesioned areas. rsFC was then computed from the preprocessed fMRI data. To derive rsFC, the brain was parcellated for the given atlases, dividing the brain into distinct ROIs. For each ROI, the average BOLD signal time series was extracted. Pearson's correlation coefficients were then calculated between the time series of all pairs of ROIs to capture the strength of time-locked connectivity between brain regions. These correlation values formed the functional connectivity matrix, which represents the rsFC features used for further analyses.

##### 2.1.3.3 FA and MD preprocessing

FA (Fractional Anisotropy) and MD (Mean Diffusivity) are quantitative metrics derived from DTI that provide insights into the microstructural properties of brain tissues and can be used to assess the integrity of white matter tracts. The diffusion data were processed following the method described in Bonilha et al. (2015). To address artifacts and noise, Gibbs artifacts removal (Kellner et al., 2015) and de-noising (Veraart et al., 2016) were performed using MRTrix tools. Spatial distortion was attenuated using FSL's TOPUP (Andersson et al., 2003) and eddy (Andersson and Sotiropoulos, 2015). FSL's dtifit was used to calculate tensors, FA, or MD. The T1 scan underwent unified normalization and segmentation using SPM12. This enabled the transformation of atlases from standard MNI to patient space. The atlases were further mapped to the native diffusion space by non-linearly warping the T1 scan to the FA and MD maps. To reduce dimensionality, the DTI connectivity of each region was averaged with its connectivity to all other regions.

#### 2.1.4 Atlases and region of interest

We segmented our images from the different modalities into regions of interest (ROIs) using the following atlases: Johns Hopkins University Atlas (JHU) (Oishi et al., 2009) with 188 ROIs, Automated Anatomical Labeling Atlas (AAL) (Tzourio-Mazoyer et al., 2002) with 108 ROIs, Brodmann Atlas (BRO) (Amunts, 2021) with 82 ROIs, and Atlas of Intrinsic Connectivity of Homotopic Areas (AICHA) (Joliot et al., 2015) with 384 ROIs. Atlases with too few regions [e.g., the Fox atlas (Fox et al., 2005) with 10 ROIs and CAT (Catani and Thiebaut de Schotten, 2008) (29 ROIs)] were excluded from our analyses because preliminary analyses showed that their low number of regions was insufficient to provide adequate predictions. For lesion modality, the value of each region was taken as the fraction of lesioned voxels (i.e., a score of “1” indicating the entire region was damaged), providing a measure of the extent of damage in specific brain regions.

We used only left-hemisphere ROIs in lesion modality because we selected participants without lesions in the right hemisphere. Therefore, this modality contains no predictive information for the right hemisphere. For rsFC, MD, SC, and FA, we used both hemispheres due to the adaptive reorganization of the brain in non-acute post-stroke patients, potentially leading to alterations in the intact hemisphere predictive of recovery.

#### 2.1.5 Connectome creation

The probabilistic white matter map of each participant, which excluded the lesion, was used as a mask to analyze the neural pathways of the tractography. By excluding white matter areas with lesions, we ensure that we include only intact neural connections in determining the brain's SC. Bedpost (Hernandez Fernandez et al., 2013) was used for fiber modeling. Subsequently, SC was quantified using probtrackx (Hernandez Fernandez et al., 2018), which assessed the SC between each region in the given atlas. For each pair of regions, the number of streamlines arriving in one region when the other was used as a seed was calculated. SC was defined as the average between the number of streamlines arriving in region A when region B was seeded and vice versa. The connectivity between the regions was corrected based on the sum of the volumes of the two regions to control for more significant regions that inherently have a higher number of streamlines than smaller regions. This resulted in a connectivity matrix of weighted connections.

### 2.2 Machine learning pipeline

We used the scikit-learn library (Pedregosa et al., 2011) to evaluate the ability of different atlas-modality-model combinations to predict behavioral scores from MRI data. In a full factorial design, we crossed four brain parcellation atlases (AAL, AICHA, BRO, JHU), five neuroimaging feature modalities (lesion, rsFC, MD, SC, and FA), and six machine learning algorithms [Linear regression (LR), random forest (RF), support vector regression (SVR), decision tree (DT), K nearest neighbors (KNN) and gradient boosting (GB)]. Separate models were trained for each behavioral outcome measure—AQ and PNT scores. SC connectome processing requires many hours per subject, so we have limited SC connectome generation to only the AICHA and JHU atlases, resulting in 216 unique combinations of atlas × modality × model × behavioral scores (Supplementary Table S3). For all analyses, we focused on ROIs defined by each atlas such that each atlas provided one feature per ROI. All region-wise imaging features (lesion, FA, MD, rsFC, SC) were vectorized into a single flattened array per subject and passed to the ML models as input. For example, using the JHU atlas (which contains 94 left-hemisphere ROIs), the lesion modality yields a 94-dimensional feature vector for each subject (each feature representing the lesion volume or proportion in one ROI). We did not apply any explicit feature selection before model fitting; instead, models were given the full set of ROI features for the specified atlas-modality, allowing them to learn which brain regions were most predictive of the outcome. We report Kruskal-Wallis H tests for each main effect and Bonferroni-corrected Dunn *post-hoc* comparisons to interpret significant differences in performance.

**Figure 2:**
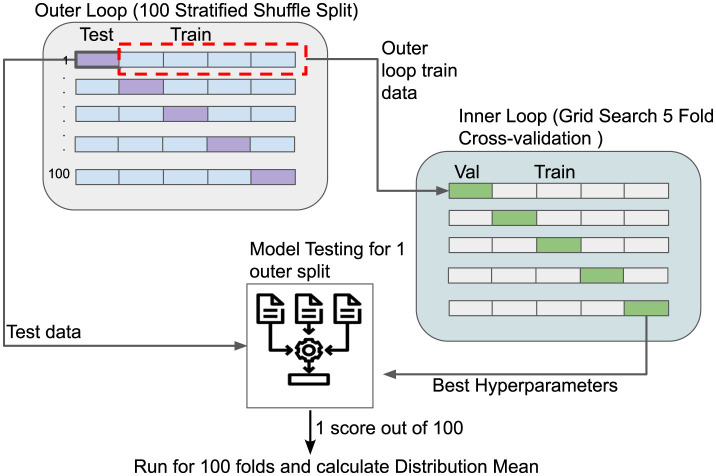
Schematic representation of the nested cross-validation scheme used to ensure no data leakage when evaluating the performances of our models.

Performance was evaluated using Pearson's correlation (*r*) between behavioral scores and predicted values in a two-level nested cross-validation with the outer loop dividing the dataset into 100, 90%–10% train-test shuffle splits (see Figure 2). The inner loop splits the remaining data into validation and training sets using a 5-fold split. The outer loop assesses in an unbiased way the performance of the model for a specific set of hyperparameters (see Table 1), while the inner loop tunes in a reproducible way those hyperparameters. The hyperparameter ranges for each algorithm were selected to balance computational efficiency with model performance, following standard practices in the ML literature. In scikit-learn, the feature_importances_ attribute of tree-based models ranks predictors by the mean decrease in impurity; larger values indicate a stronger contribution to predictive performance.

**Table 1:** Hyperparameters explored for different ML models.

**Model**	**Hyperparameters**
DT	• criterion: squared_error, friedman_mse, absolute_error, poisson • splitter: best, random • max_depth: 1, 3, 5 • min_samples_leaf: 1, 2 • min_weight_fraction_leaf: 0.1, 0.2 • max_features: log2, sqrt, None
GB	• loss: ls, absolute_error, huber, quantile • learning_rate: 0.05, 0.25, 0.50, 1 • criterion: friedman_mse, squared_error • max_features: log2, sqrt
KNN	• criterion: squared_error, friedman_mse, absolute_error, poisson • splitter: best, random • max_depth: 1, 3, 5 • min_samples_leaf: 1, 2 • min_weight_fraction_leaf: 0.1, 0.2 • max_features: log2, sqrt, None
LR	• fit_intercept: True, False • copy_X: True, False • positive: True, False
RF	• n_estimators: 100 • max_features: sqrt, log2, None • max_depth: 15, 20 • min_samples_leaf: 4, 8, 16 • bootstrap: True, False • criterion: squared_error, absolute_error, poisson
SVR	• cache_size: 100, 200 • degree: 2, 4 • gamma: scale, auto • kernel: linear, poly, rbf, sigmoid • shrinking: True, False • verbose: True, False

Performance was assessed using these parameters in the outer loop of nested cross-validation.

## 3 Results

**Figure 3:**
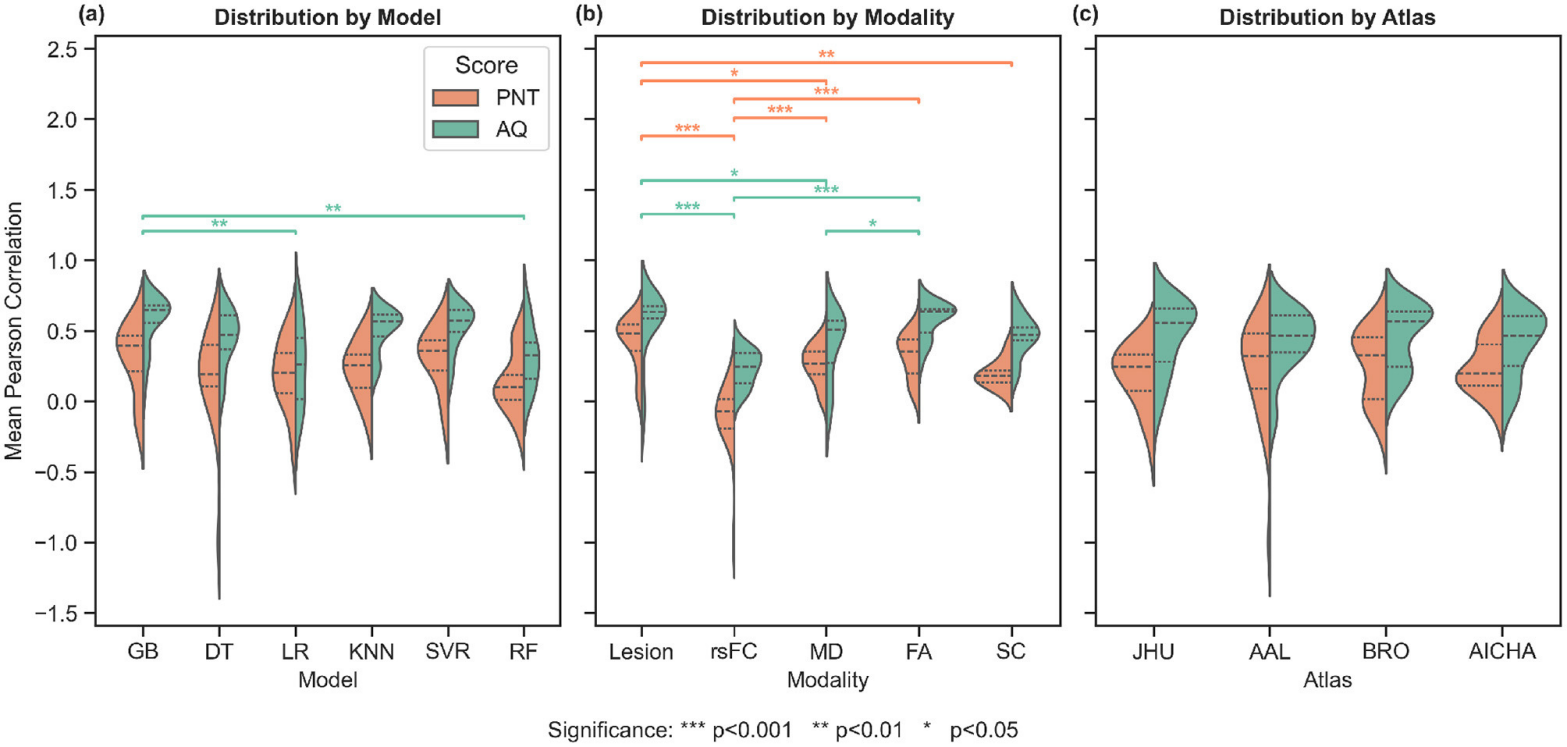
Prediction-accuracy violins (shared *y*-axis) for AQ (orange) and PNT (green). Left: by model **(a)**. Middle: by modality **(b)**. Right: by atlas **(c)**. Dashed lines indicate medians and quartiles. These distributions also include non-significant results. Refer to Supplementary Figure S2 for heatmaps showing individual results.

We performed a full factorial analysis of prediction accuracy (Pearson's *r* between predicted and actual behavioral scores) with factors **ML Model**, **Neuroimaging Modality**, and **Atlas**. Figure 3 presents violin plots comparing the distribution of correlation scores (shared *y*-axis) for AQ and PNT across each factor level. Overall, AQ predictions were more accurate than PNT (a higher median *r* across all conditions), but the patterns of factor effects were broadly similar. Among the three factors, imaging modality had the strongest influence on model performance, whereas model algorithm and atlas had more modest effects (especially for PNT). A complete breakdown of additional results is provided in the Supplementary material. Supplementary Figure S2 presents heatmaps of subscore correlations for every model. Multimodal outcomes and model comparisons are summarized in Supplementary Table S2. Supplementary Figure S1 plots linear regressions between each of the four WAB-R subscores (fluency, comprehension, repetition, and naming) and PNT prediction, while Supplementary Table S3 outlines how the 216 combinations were derived.

### 3.1 Machine learning model effects

For AQ prediction, significant differences were observed across machine learning models (Kruskal-Wallis: *H*(5)=23.22, *p* < 0.001; Figure 3a). *Post-hoc* Dunn tests with Bonferroni correction revealed that GB significantly outperformed both LR (*p* < 0.01) and RF (*p* < 0.01). In contrast, model selection did not significantly influence PNT prediction accuracy (*H*(5) = 8.94, *p*>0.05).

### 3.2 Neuroimaging modality effects

Imaging modality demonstrated the strongest influence on prediction performance. For AQ scores, modality effects were highly significant (*H*(4) = 43.48, *p* < 0.001; Figure 3b), with *post-hoc* tests revealing significant differences between FA vs. rsFC (*p* < 0.001), FA vs. MD (*p* < 0.05), rsFC vs. lesion (*p* < 0.001), and lesion vs. MD (*p* < 0.05). Modality significantly affected PNT prediction (*H*(4) = 60.31, *p* < 0.001), with significant differences between SC vs. lesion (*p* < 0.01), FA vs. rsFC (*p* < 0.001), rsFC vs. lesion (*p* < 0.001), rsFC vs. MD (*p* < 0.001), and lesion vs. MD (*p* < 0.05).

### 3.3 Atlas parcellation effects

Atlas selection did not significantly influence prediction accuracy for either AQ (*H*(3) = 1.73, *p*>0.05; Figure 3c) or PNT scores (*H*(3) = 1.66, *p*>0.05).

### 3.4 Notable model-modality-atlas combination

**Figure 4:**
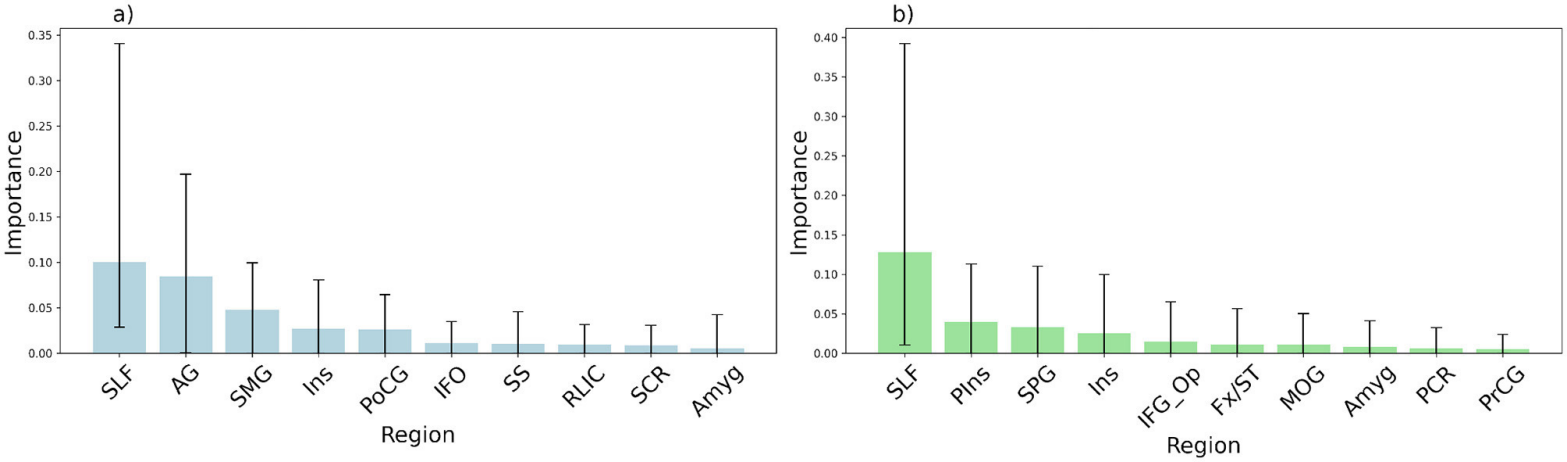
Feature importance for the top 10 regions identified using RF. **(a)** Feature importance for AQ scores across different brain regions, with the bars representing the mean importance values and the whiskers indicating 95% bootstrapped confidence intervals. **(b)** As in panel **(a)**, but for PNT scores. Refer to Table 3 for abbreviations.

**Figure 5:**
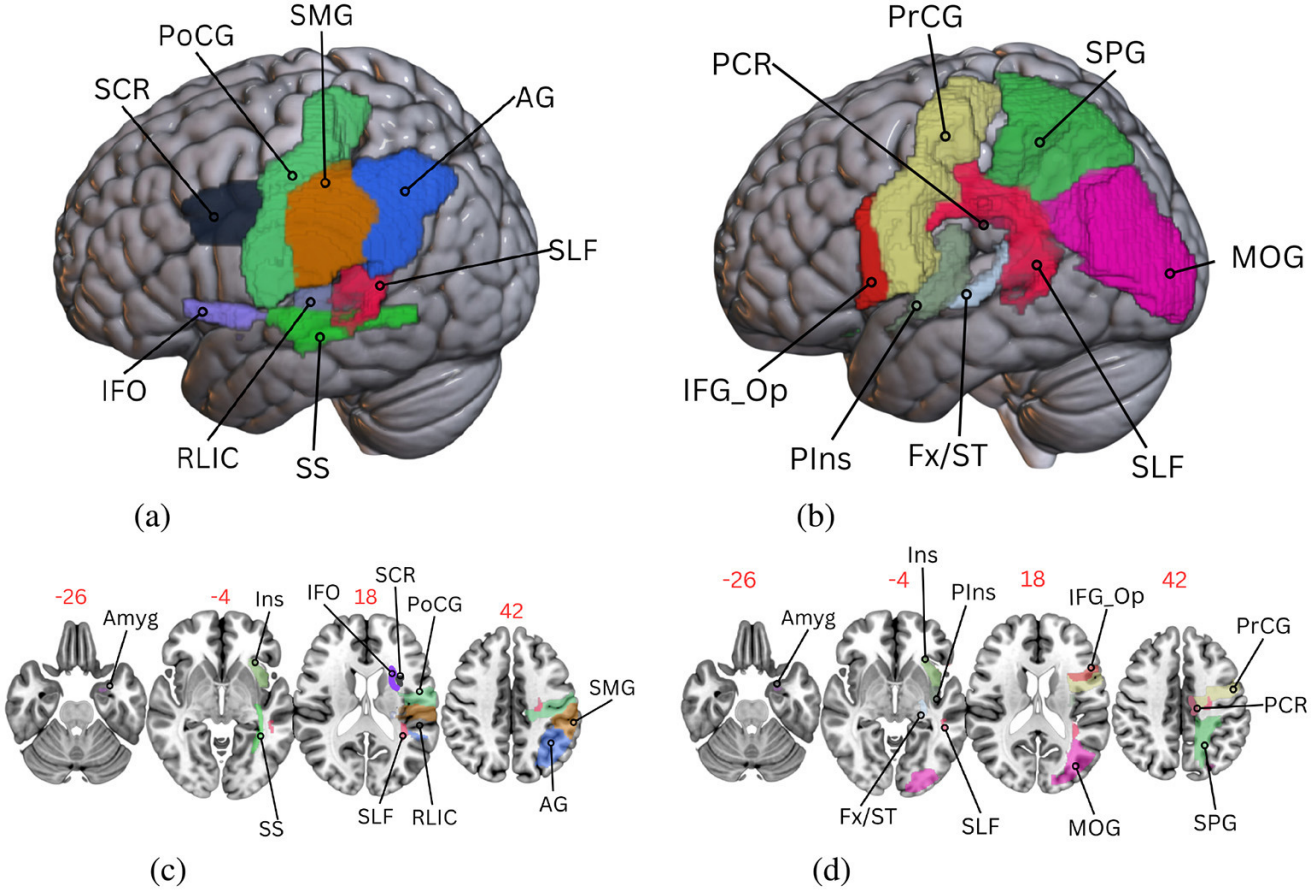
Visualization of the most important brain regions for aphasia in the MNI152 space using the JHU atlas, as identified by the Random Forest algorithm applied to both AQ and PNT. **(a)** AQ JHU surface regions. **(b)** PNT JHU surface regions. **(c)** AQ JHU axial regions. **(d)** PNT JHU axial regions.

Despite RF showing lower overall performance across conditions, the specific combination of RF with lesion data and JHU atlas achieved the highest correlation for AQ prediction (*r* = 0.73 ± 0.09) (Table 2) in our experiments, exceeding previously reported benchmarks in the literature (Yourganov et al., 2016). This finding suggests that while RF may not be the optimal model when averaged across conditions, it exhibits excellent performance with this specific combination of lesion data and JHU atlas for predicting aphasia severity. The fact that this correlation is the mean value from a bootstrapped distribution rather than a point estimate supports the robustness of this result. Further, GB also exhibited a 0.72 correlation for the same condition, corroborating that high correlations for this condition are not only due to a peculiarity of the RF model (see Supplementary Figure S3 for the top ten feature-importance plots for GB). To better understand RF's strong performance, we examined the feature importance scores from the RF model. The top 10 brain regions for AQ and PNT predictions are shown in Figure 4, with mean importance values and 95% bootstrapped confidence intervals (see Table 3 for region abbreviations). To visualize their spatial distribution, Figure 5 shows the most important regions in MNI152 space using the JHU atlas, with surface and axial views for both AQ and PNT predictions.

**Table 2:** Pearson's correlation coefficients (mean ± standard deviation) between actual and predicted AQ and PNT scores (current study: JHU atlas and lesion modality) along with associated *p*-values (bootstrapped with replacement, 100 iterations).

**Previous studies**				
**Study**	**AQ (** ***r*** **)**	***p*** **-value (AQ)**	**PNT (** ***r*** **)**	***p*** **-value (PNT)**
Yourganov et al. (2016)	0.69 (*N* = 90)	< 0.01	-	-
Kristinsson et al. (2020)	0.44 (*N* = 116)	< 0.01	-	-
	**Current study**			
**ML model**	**AQ (*****N*** = **238)**	***p*** **-value (AQ)**	**PNT (*****N*** = **191)**	***p*** **-value (PNT)**
Decision tree	0.69 ± 0.07	< 0.01	0.29 ± 0.15	0.05
Gradient boosting	0.72 ± 0.06	< 0.01	0.48 ± 0.11	< 0.01
K nearest neighbors	0.62 ± 0.07	< 0.01	0.29 ± 0.15	0.04
Linear regression	0.24 ± 0.21	0.19	0.07 ± 0.16	0.30
**Random forest**	0.73 ± 0.09	< 0.01	0.46 ± 0.11	< 0.01
Support vector regression	0.67 ± 0.07	< 0.01	0.38 ± 0.11	< 0.01

Results from prior studies are included for comparison.

**Table 3:** Functional localization of brain regions in the left hemisphere, as identified by Random Forest feature importance analysis.

**Region (Abbreviation)**	**Measures**
Superior longitudinal fasciculus left (SLF)	AQ, PNT
Angular gyrus left (AG)	AQ
Insular left (Ins)	AQ, PNT
Superior corona radiata (SCR)	AQ
Amygdala (Amyg)	AQ, PNT
Supramarginal gyrus left (SMG)	AQ
Retrolenticular part of internal capsule left (RLIC)	AQ
Inferior fronto-occipital fasciculus left (IFO)	AQ
Postcentral gyrus left (PoCG)	AQ
Sagittal stratum left (SS)	AQ
Fornix (cres) / Stria terminalis (Fx/ST)	PNT
Precentral gyrus left (PrCG)	PNT
Superior parietal gyrus left (SPG)	PNT
Posterior insula left (PIns)	PNT
Posterior corona radiata left (PCR)	PNT
Inferior frontal gyrus pars opercularis left (IFG_Op)	PNT
Middle occipital gyrus left (MOG)	PNT

The table lists the region abbreviation and the measures (AQ or PNT) for which that region was predictive.

### 3.5 Effect of combining modalities

We systematically tested various combinations of lesion, diffusion (FA, MD), and rsFC features, which could boost predictive accuracy. However, the integration of these modalities led to a decline in performance. Models that used only diffusion and rsFC features with RF (e.g., FA + rsFC: *r* = 0.329; FA + MD: *r* = 0.202) achieved lower predictive accuracy compared to models that included lesion features. When lesion features were combined with individual modalities, performance modestly improved (e.g., lesion + FA: *r* = 0.264; lesion + MD: *r* = 0.249; lesion + rsFC: *r* = 0.189). Further, adding two modalities alongside lesion maps yielded slightly higher correlations (e.g., lesion + FA + fMRI: *r* = 0.375; lesion + MD + rsFC: *r* = 0.319; lesion + FA + MD: *r* = 0.308). The best performance was observed when all modalities were combined (lesion + FA + rsFC + MD: *r* = 0.358), although this remained lower than the lesion-only model. Full details of all combinations and corresponding results are provided in Supplementary Table S2.

### 3.6 ROI identification using machine learning

Figure 4 illustrates the distribution of feature importance of the ten most important features (i.e., in our case, brain regions) for predicting PNT and AQ. In Figure 5, we show the most predictive regions of the JHU parcellation in the MNI152 space. The identified top features from the RF model for AQ and PNT are provided in Table 3. Although these are the most important regions, others may also contribute to language outcomes in people with aphasia. The feature correlation matrix revealed the presence of significant multicollinearity (unpublished results; we did not include this matrix in supporting information because its 188 × 188 size results in unreadable labels when fit within the limit of a paper sheet); several pairs of variables are highly correlated with one another, exhibiting an *r* > 0.8. For instance, in the left hemisphere, the Amygdala shows a particularly high correlation with behavioral scores, as do the Caudate Nucleus and Globus Pallidus. Nevertheless, the feature importance analysis and prior knowledge from the literature on key regions in aphasia suggest that the RF model uses relevant regions to predict language outcomes. The identified regions are biologically plausible and clinically relevant, suggesting that such modeling could be useful to support LSM.

## 4 Discussion

In our benchmarking study, the choice of neuroimaging modality was the dominant factor influencing aphasia outcome prediction accuracy, exceeding the impact of the ML model or the brain atlas used. In particular, models built on lesion data and FA features significantly outperformed those using other imaging modalities. Ensemble tree-based algorithms (GB) produced higher median correlations than simpler learning models (LR, DT, or KNN). By contrast, brain atlas selection had no significant effect on performance. We also observed that PNT scores were markedly harder to predict than the AQ. The best prediction for overall aphasia severity (AQ) reached a higher correlation (*r* = 0.73; using RF) than for naming ability (PNT, *r* = 0.48; using GB), despite these behavioral scores being highly correlated with one another (in our dataset, *r* = 0.89). This disparity might partly stem from the smaller sample size for PNT (*N* = 191) compared to AQ (*N* = 238), providing less data for ML training and, therefore, lower performance for PNT prediction. As a composite score, the WAB AQ also smooths out variability across tasks. In contrast, the PNT, focusing purely on naming, could be more sensitive to specific lesion patterns that are harder to capture with features based on atlases' ROIs.

Neuroimaging modality emerged as the most critical determinant of model performance, underscoring the importance of informative brain damage and connectivity biomarkers. Models based on stroke lesion features yielded the highest accuracies, closely followed by those using FA. This result aligns with longstanding clinical observations that lesion location and volume strongly predict chronic aphasia severity (Billot et al., 2022a). A large lesion encroaching on critical language zones (e.g., left perisylvian cortex or underlying white matter) will typically produce severe impairment, which makes lesion-based features highly informative for predicting composite scores like the AQ. LSM studies have found significant correlations between lesion extent and language deficits (Billot et al., 2022a). Our results reaffirm that simply knowing where and how much tissue is destroyed provides a robust basis for outcome prediction. FA added nearly equivalent value, showing that the micro-structural integrity of white-matter tracts is almost as informative as gray-matter loss. The shortfall of rsFC is likely due to (i) high measurement noise, (ii) limited sample size relative to feature dimensionality, and (iii) the fact that much of the functional disruption can already be inferred from structural damage. Clinically, these results endorse a pragmatic imaging protocol: *structural T1/T2 + DTI* provides most of the prognostic information, whereas resting-state fMRI may not justify its cost for baseline severity estimation.

Regarding the choice of ML models, most performed comparably and were significantly correlated with language proficiency measures. Tree-based ensembles (i.e., GB, RF) captured non-linear interactions and were robust to multicollinearity among ROI features, explaining their ~5–10% advantage in median *r* over simpler models. While linear regression occasionally approached ensemble performance in low-dimensional settings, its susceptibility to highly correlated predictors makes it unreliable. The practical implication is straightforward: high feature dimensionality and non-linear relationships favor an ensemble learner (GB, RF) or similarly expressive model. However, LR significantly underperformed compared to the other models, suggesting that this model is not well-suited for this task. Multicollinearity is likely to limit the performance of LR models notably. Multicollinearity can lead to inflated standard errors, making it challenging to discern the true effect of independent variables. Additionally, the high degree of intercorrelation may increase the variance of the coefficient estimates and make the model more sensitive to small changes in their values.

We settled on RF for various reasons. As with other well-performing models (see Table 2), RF predictions were significantly more accurate than chance. Neuroimaging benchmarks show that impurity-based and permutation-based rankings converge when random forests are built with sufficient depth and tree count (e.g., McPartland, 2024); our configuration of 100 trees with max_depth=20 meets these conditions while remaining computationally tractable. For SC, this was true even when connections involving lesioned regions were omitted from the analysis. This suggests that ML analysis can effectively mitigate the spatial bias toward areas more likely to be lesioned due to their location relative to blood vessels. RF is also advantageous because it provides a convenient way to assess feature importance. We benefited from this technical capability to determine which brain regions contribute most to aphasia symptoms. Identified brain regions (see Table 3) are known to be associated with language-related difficulties (Dronkers et al., 2004; Faroqi-Shah et al., 2014; Ouden et al., 2019), such as difficulties in speech comprehension, production, reading, writing, and object naming. These results support the capability of this approach to identify key brain regions in aphasia from lesion mapping.

The choice of brain atlas had a much smaller effect on prediction performance. Five of the six atlases tested yielded very similar accuracies, with differences of only a few percent in correlation. This robustness to atlas choice suggests our findings are not tied to an idiosyncratic brain partition; the signal can be captured in multiple atlas frameworks. Following preliminary analyses, we rejected the FOX atlas from our comparison as it yielded systematically worse performance, possibly because it included only a very small number of regions (*N* = 10), which poorly align with the functional anatomy of language. From a methodological standpoint, while many atlases work interchangeably, one should avoid atlases that might not capture the regions of interest for a given clinical question.

We also tested whether integrating lesion maps with diffusion (FA, MD) and rsFC features could boost predictive accuracy. In practice, naïvely concatenating these modalities inflated our feature set relative to sample size, resulting in over-fitting and only marginal or negative performance changes (e.g., lesion + FA + rsFC: *r* = 0.38 vs. lesion alone: *r* = 0.73, Supplementary Table S2). This suggests that in chronic post-stroke aphasia, the structural lesion signature contains the lion's share of prognostic information, and successful multimodal fusion will require rigorous feature-selection or dimensionality-reduction methods rather than simple feature stacking.

The primacy of lesion and white matter integrity measures reinforces that stroke-induced aphasia is predominantly a disorder of structural brain damage. A machine learning model drawing only on lesion pattern can achieve a correlation of 0.7 with actual severity, approaching clinical utility. Damage to any region in Table 3 can lead to language impairments commonly associated with aphasia severity (Galantucci et al., 2011; Ivanova et al., 2016; Griffis et al., 2017). In particular, SLF emerges as an essential feature in the RF model, showing substantial impact and its role in enhancing PNT and AQ prediction performance. SLF is a large bundle of fibers that connects the frontal areas with other areas of the ipsilateral hemisphere, notably the parietal areas (Janelle et al., 2022). By connecting the frontal regions involved in speech production, phonology, and domain-general executive functions with posterior areas related to verbal short-term memory and semantics, SLF plays a pivotal role in multiple language functions (Bernal and Ardila, 2009). Similarly, other regions identified here are part of the classic “language cortex” (see Desai and Riccardi, 2021; Kemmerer, 2022 for reviews). The AG is a heteromodal association zone involved in a variety of language tasks, particularly those related to semantic processing (Desai et al., 2023; Riccardi et al., 2022; Riccardi and Desai, 2022; Binder and Desai, 2011). The MOG is usually not considered part of the traditional “language network”. Still, it can be considered an early part of the ventral language stream and has been implicated in tasks related to picture description (Riccardi et al., 2024), likely reflecting its role in visual processing of objects and visual semantics (Fridriksson et al., 2016, 2018; Hickok and Poeppel, 2007). SMG is involved in short-term auditory and verbal memory and phonology (Hartwigsen et al., 2010; Deschamps et al., 2014), two vital functions for the repetition and production tasks tested by WAB-R. Also pertaining to production, the insula and ventral precentral gyrus were identified as important features for both AQ and PNT, which aligns with research demonstrating that disruption of these areas is related to disrupted speech production and fluency (Riccardi et al., 2023; Blackett et al., 2022; Ackermann and Riecker, 2010; Fridriksson et al., 2015).

There are a few limitations to consider in this study. The DTI and functional connectivity of each region were reduced by averaging its connectivity to all regions to reduce dimensionality. Hence, information about the connectivity between individual pairs of brain regions is lost. The lower performance of ML models using SC and rsFC may be partly due to this loss of information about the connectivity structure. Also, although this study used a relatively large dataset for MRI and aphasia studies, many ML models typically require big corpora for reliable training and estimation. Ivanova et al. (2021) suggested that the spatial accuracy of LSM plateaus at about *N* = 130, with little to no gain associated with further increasing the sample sizes. However, this may not hold when many features from multiple modalities are used.

We also recognize that impurity-based importance is only one of several ways to interpret model weights. We nevertheless retained the native RF metric in results included in Section 3.6 for two practical reasons. First, RF was the top-performing learner in our grid search (highest bootstrapped mean r across the full 216, atlas × modality × model × scores combinations; Supplementary Figure S2), so it is the natural source for *post-hoc* interpretation. Second, impurity ranks are produced “for free” by the trained RF. In contrast, model-agnostic alternatives—permutation, Shapley Additive Explanations (SHAP) (Lundberg and Lee, 2017), or leave-one-feature-out would have raised computational cost by two to three orders of magnitude when applied to the largest atlas (384 ROIs) over 100 bootstrap folds and ~1.3 million trees.

An important avenue for future work is to a) investigate how the integrity of specific brain regions contributes to the prediction of aphasia severity or type, as reflected across different neuroimaging modalities; b) assess whether the predictive value of these brain regions varies depending on the neuroimaging modality used (e.g., structural MRI, DTI, or resting-state fMRI). The resulting knowledge can further inform targeted interventions and therapeutic strategies for individuals with language disorders resulting from brain lesions. Future work could explore more advanced ML solutions, such as Neurosymbolic AI (infusing expert knowledge in ML models beyond the initial data representation), convolutional autoencoders, or spatially constrained autoencoders. This approach balances dimensionality reduction and spatial fidelity, ensuring that the spatial intricacies crucial for our application are retained. Additionally, exploring the stability and consistency of the feature importance rankings across different datasets would contribute to the robustness and generalizability of the findings.

## 5 Conclusion

This study employed a comprehensive factorial benchmarking of imaging modalities, machine learning algorithms, and brain atlases for predicting chronic post-stroke language outcomes. Key findings indicate that imaging modality, particularly structural measures such as lesion load and diffusion tensor fractional anisotropy, outweighs the influence of the choice of machine learning model or brain atlas in predicting aphasia severity. Among the models, ensemble methods like RF and GB provided the highest predictive accuracy. The choice of atlas showed minimal impact on performance, except when using overly coarse parcellations. These insights underline the potential of using structural MRI combined with advanced machine learning techniques to develop clinically viable tools for aphasia prognosis, highlighting areas for future research to enhance model validation and address limitations noted in sample size and diversity.

## Data Availability

The datasets presented in this study can be found in online repositories. The names of the repository/repositories and accession number(s) can be found at: https://openneuro.org/datasets/ds004884/versions/1.0.1.
