# Supplementary material for: Benchmarking machine learning models in lesion-symptom mapping for predicting language outcomes in stroke survivors
Source: Front Neuroimaging. 2025 May 30;4:1573816. doi: 10.3389/fnimg.2025.1573816 (PMC12163030; doi:10.3389/fnimg.2025.1573816)
Supplement: Supplementary file 1 [file Data_Sheet_1.pdf]

Supplementary Material :

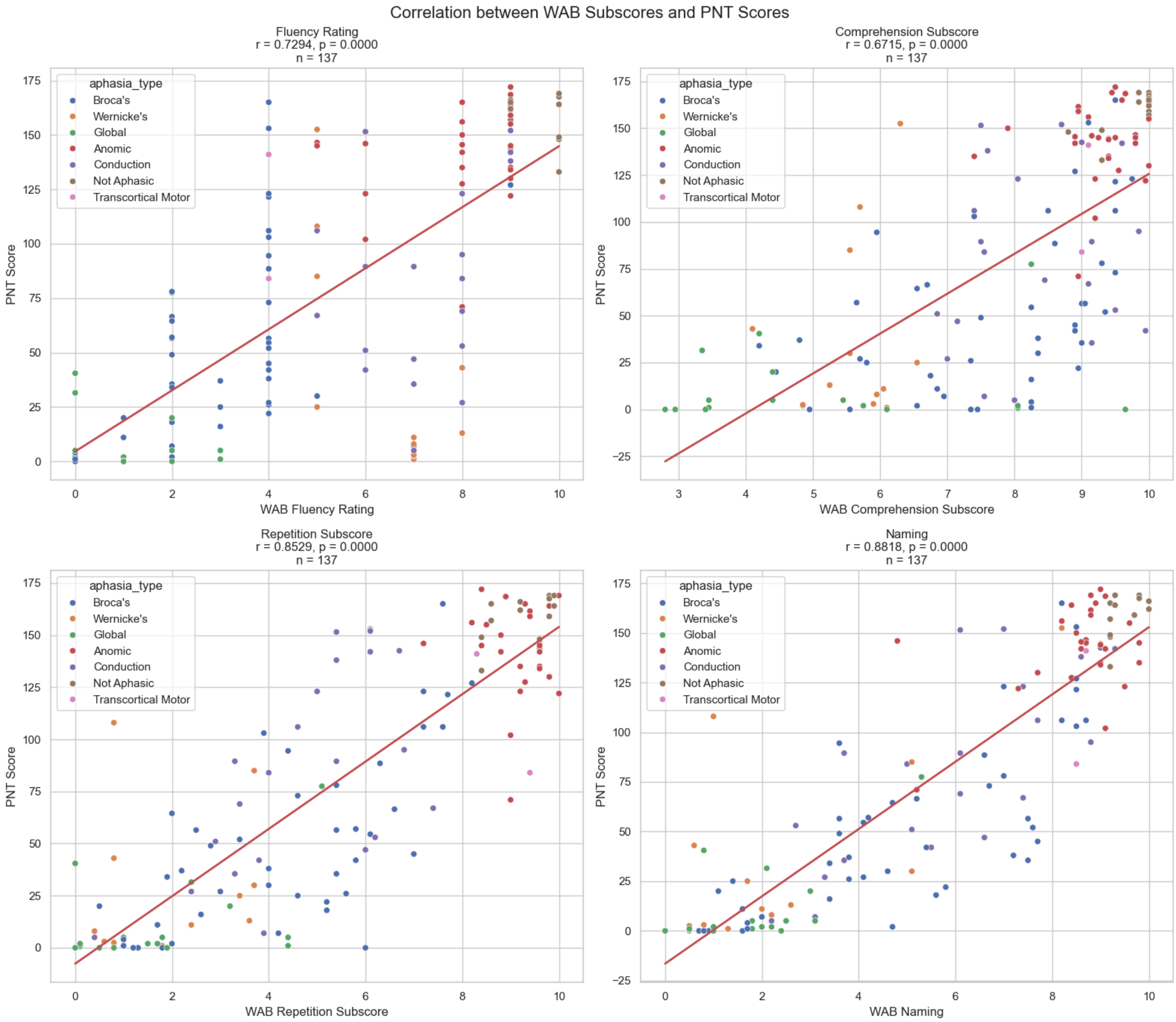

Figure S1: Comparison of AQ subscores and PNT by aphasia type.

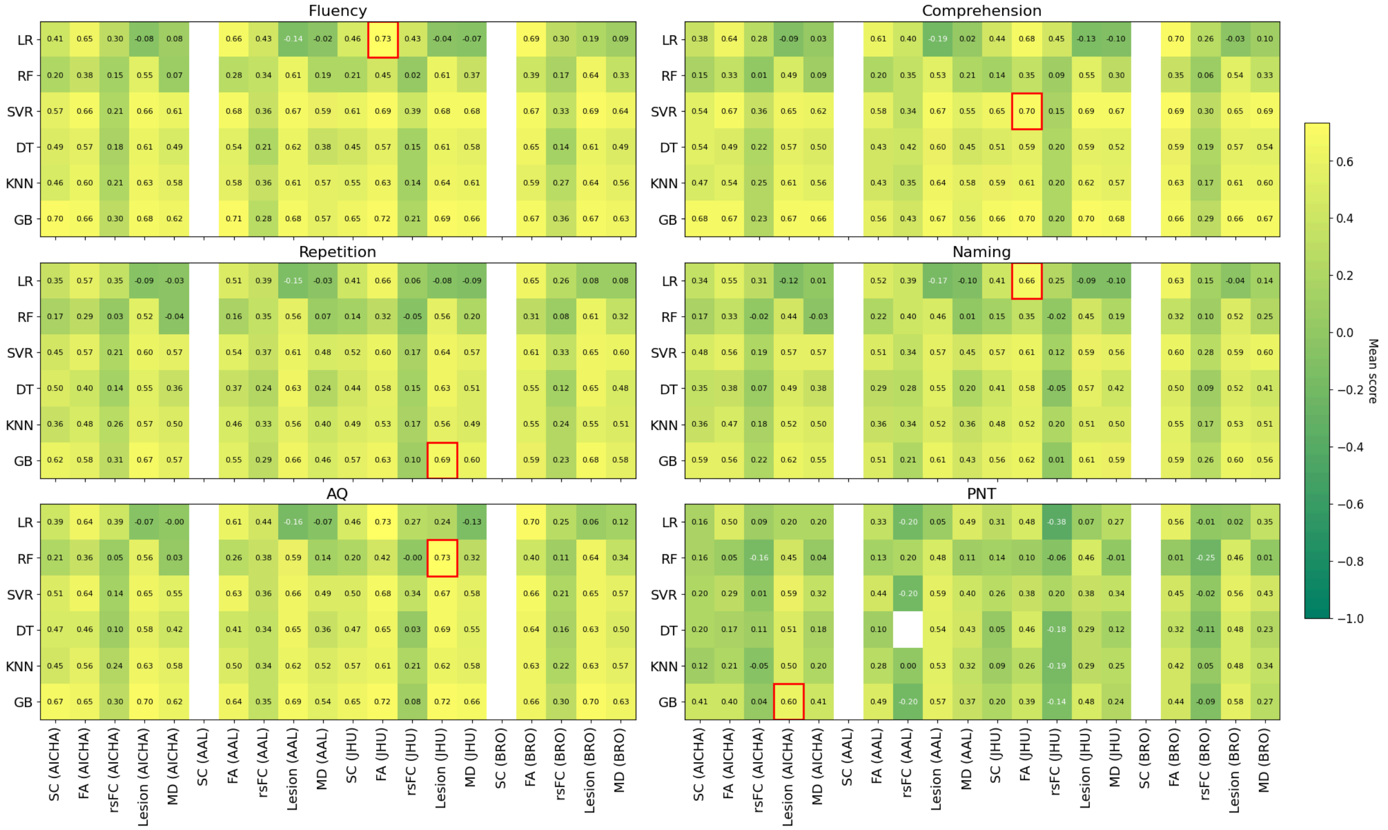

Figure S2: Heatmaps of mean predictive performance (masked by $p < 0.05$) across six language subscores (FLUENCY, COMPREHENSION, REPETITION, NAMING, AQ, and PNT), stratified by model type (rows) and modality–atlas combinations (columns). Each cell displays the mean score, with red borders highlighting the best-performing atlas for each model in a given subscore. Color intensity indicates performance (lighter = higher mean score). The models include LR, RF, SVR, DT, KNN, and GB; modalities include SC, sFC, MD, FA, and lesion; atlases include AAL, AICHA, BRO, and JHU.

Subscore	AAL	AICHA	BRO	JHU
**AQ**	0.592	0.558	0.636	0.730

**Comprehension**	0.526	0.488	0.545	0.547
**Fluency**	0.609	0.547	0.636	0.613
**Naming**	0.456	0.437	0.515	0.448
**Repetition**	0.564	0.523	0.607	0.559
**PNT**	0.477	0.449	0.460	0.460

Table S1. Mean performance of the Random Forest model on the lesion modality across six atlases (AAL, AICHA, BRO, and JHU) for each subscore.

ML model	fa_rsFC	fa_md	lesion_fa	rsFC_md	rsFC_lesion	lesion_md	fa_rsFC_md	lesion_fa_fmri	lesion_md_rsFC	lesion_fa_md	lesion_fa_rsFC_md
RF	0.329	0.202	0.264	0.245	0.18975	0.249	0.293	0.375	0.319	0.308	0.358
KNN	-0.118	0.227	0.132	-0.012	0.020	0.063	-0.118	0.113	0.096	0.132	0.113
DT	-0.351	-0.699	-0.493	-0.114	-0.66	-0.237	-0.022	0.150	-0.360	-0.304	-0.422
SVR	0.014	-0.114	-0.002	0.012	0.005	-0.001	0.013	0.027	0.026	-0.008	0.027
LR	-0..612	-2.196	-0.440	-0.119	-0..27	-0.280	-0.614	-0.584	-0.193	-0.517	-1.584
GB	0.293	0.210	0.369	0.105	0.09	0.208	0.193	0.351	0.314	0.252	0.355

Table S2 Performance of ML models (RF, KNN, DT, SVR, LR, and GB) across different feature combinations (fa_rsFC, fa_md, lesion_fa, rsFC_md, lesion_md, etc.), showcasing the model-specific impact on predicting outcomes.

Atlas	Modalities available	#conds = (modalities × 6 models × 2 scores)
AICHA	SC, rsFC, MD, FA, lesion (5)	5 × 6 × 2 = 60
JHU	SC, rsFC, MD, FA, lesion (5)	5 × 6 × 2 = 60
AAL	rsFC, MD, FA, lesion (4)	4 × 6 × 2 = 48
BRO	rsFC, MD, FA, lesion (4)	4 × 6 × 2 = 48
**Total**		**216**

Table S3 : **The table summarizes for each available modality the number of atlases, model×atlas trios, subscores per trio, and subscore-cells.**

.

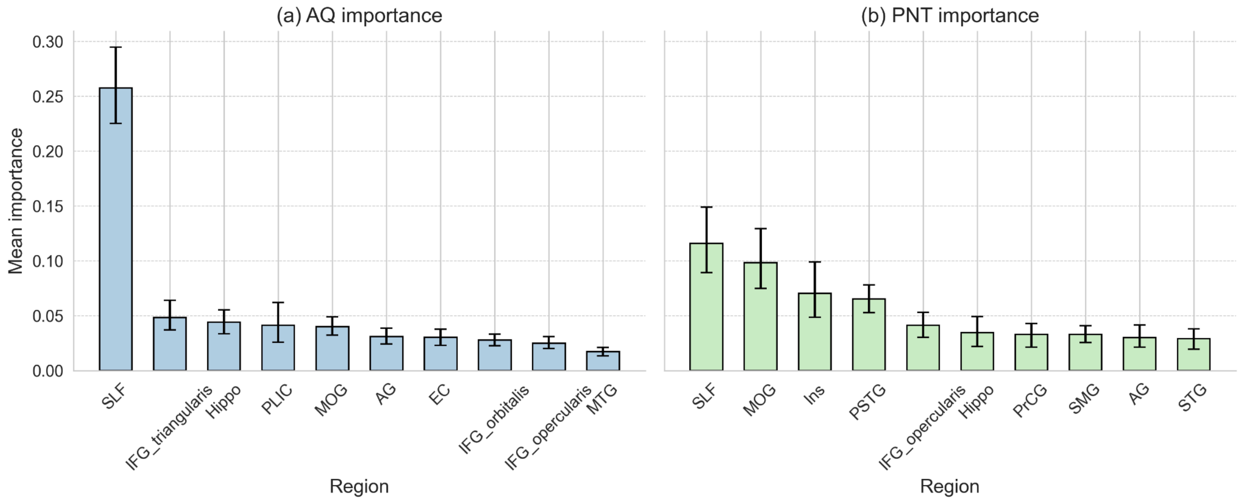

Figure S3: Bootstrapped Gradient Boosting feature importances for lesion predicting (a) AQ and (b) PNT, showing mean importance (95% CI) with regions on the x-axis.
